# Fluctuation–dissipation relations far from equilibrium: a case study

**DOI:** 10.1039/d1sm00521a

**Published:** 2021-06-07

**Authors:** Gerhard Jung, Friederike Schmid

**Affiliations:** Institut für Theoretische Physik, Universität Innsbruck Technikerstraße 21A A-6020 Innsbruck Austria gerhard.jung@uibk.ac.at; Institut für Physik, Johannes Gutenberg-Universität Mainz 55099 Mainz Germany friederike.schmid@uni-mainz.de

## Abstract

Fluctuation–dissipation relations or “theorems” (FDTs) are fundamental for statistical physics and can be rigorously derived for equilibrium systems. Their applicability to non-equilibrium systems is, however, debated. Here, we simulate an active microrheology experiment, in which a spherical colloid is pulled with a constant external force through a fluid, creating near-equilibrium and far-from-equilibrium systems. We characterize the structural and dynamical properties of these systems, and reconstruct an effective generalized Langevin equation (GLE) for the colloid dynamics. Specifically, we test the validity of two FDTs: The first FDT relates the non-equilibrium response of a system to equilibrium correlation functions, and the second FDT relates the memory friction kernel in the GLE to the stochastic force. We find that the validity of the first FDT depends strongly on the strength of the external driving: it is fulfilled close to equilibrium and breaks down far from it. In contrast, we observe that the second FDT is always fulfilled. We provide a mathematical argument why this generally holds for memory kernels reconstructed from a deterministic Volterra equation for correlation functions, even for non-stationary non-equilibrium systems. Motivated by the Mori–Zwanzig formalism, we therefore suggest to impose an orthogonality constraint on the stochastic force, which is in fact equivalent to the validity of this Volterra equation. Such GLEs automatically satisfy the second FDT and are unique, which is desirable when using GLEs for coarse-grained modeling.

## Abstract 2

We investigate by simulations and theory the validity of different fluctuation–dissipation theorems (FDT) in non-equilibrium systems and find that the second FDT is never fundamentally violated.

## Graphical abstract

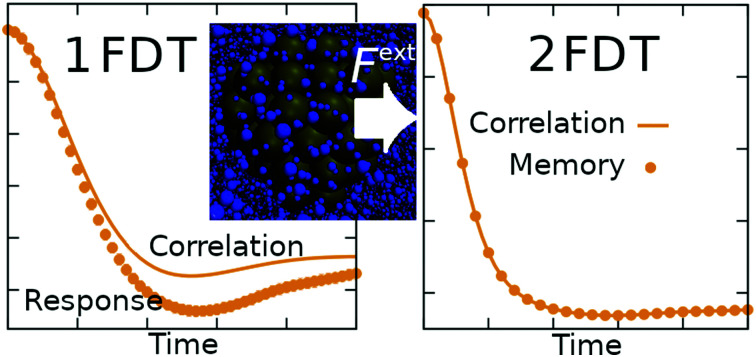

## I. Introduction

Fluctuation–dissipation theorems (FDTs) combine the distinct worlds of “thermal fluctuations” and “dissipative response” and have become a cornerstone of statistical physics^1–7^ with many applications in condensed matter physics^8–12^ (just to name a few). In the literature several distinct forms of FDTs appear. The most common one is derived from linear response theory and relates the non-equilibrium response function of an observable to the relaxation of equilibrium fluctuations. This relation corresponds to Onsager's hypothesis, stating that a system cannot differentiate between forced and spontaneous fluctuations.^2^ In the following this relation will be referred to as first fluctuation–dissipation relation 1FDT. Another FDT appears in generalized Langevin equations and connects the systematic, friction interactions in the system, described by the memory kernel, with the coloured thermal noise. We refer to this relation as second fluctuation–dissipation relation 2FDT.

For equilibrium systems, the FDTs can be rigorously derived within linear response theory,^3,13^ their validity in non-equilibrium situations has, however, been extensively and controversially discussed in the literature.^9–11,14–24^ Outside the linear response regime, these theorems should therefore be rather seen as unproven “relations”.^25^ One reason for the controversies might be that an apparent violation of the FDT could be caused by an incorrect generalization of the equilibrium FDT to non-equilibrium systems. For example, in the case of active microrheology, it has been shown that close to equilibrium a FDT can be recovered when considering an additive correction accounting for the local mean velocity of the particle.^6,17,26^ For our system this implies that the 1FDT is valid in the Galilean reference frame that moves with the average velocity of the colloid (which will be called “colloid frame” in the following). This can be intuitively understood from Onsager's hypothesis according to which the relaxation of forced fluctuations in the non-equilibrium steady-state should be related to spontaneous fluctuations around this non-equilibrium state. Other situations that can lead to apparent violation of the 1FDT have been discussed in.^9,27,28^ An intuitive Gedanken-experiment is a system in which two thermostats with different temperature act on different degrees of freedom of a particle (*i.e.* in different dimensions) and a cross correlation exists between these degrees of freedom. Such systems appear to violate the 1FDT, however, differences between response and fluctuations can be directly related to the temperature difference and the strength of the cross correlations.^27,28^

Discussions of the 2FDT in non-equilibrium systems have so far been scarce in the literature. From a theoretical perspective, the situation is clear for dynamical systems with unitary time evolution. This includes classical and quantum mechanical Hamiltonian systems, but also quasi-Hamiltonian systems such as Molecular Dynamics models that include Nose–Hoover thermostats. Applying the Mori–Zwanzig formalism, one can then exactly rewrite the microscopic equations of motion in terms of a GLE for coarse-grained variables, and derive an 2FDT for stationary^29^ or non-stationary^30^ systems without any assumptions – apart from the requirement that the space of dynamical variables forms a Hilbert space (and thus an inner product is defined). In non-Hamiltonian systems, however, the validity of the 2FDT has been questioned, and in fact several recent papers have suggested violation of the 2FDT.^19–23,31,66^ It is therefore desirable to understand potential origins for the violation of FDTs in non-equilibrium systems.

In the present paper, we investigate the validity of the fluctuation–dissipation relations in non-equilibrium steady-states using the example of active microrheology.^32^ For this purpose we study the linear and non-linear response of a colloid immersed in a fluid described by dissipative particle dynamics (DPD)^33,34^ to an externally applied driving force. To evaluate the FDTs we analyze the properties of the tracer particle in the colloid frame in detail. We reconstruct the memory kernel, which allows us to determine the coloured thermal noise. In this way we can not only validate the 2FDT but also extract the noise distribution which shows an unexpected, asymmetric non-Gaussian behaviour for systems far away from equilibrium (*i.e.* pulling forces outside the linear response regime). Furthermore, we observe an apparent violation of the 1FDT far away from equilibrium, which we interpret in terms of the aforementioned two-thermostat model.

Our manuscript is organized as follows. In Section II we introduce in detail the two fluctuation–dissipation relations that will be studied in this work and present some novel results on the 2FDT in non-equilibrium and possibly even non-stationary systems. We then describe the simulation model and analysis techniques, including the reconstruction of the memory kernel and determination of the noise in Section III. Afterwards, in Section IV, we analyze the response of the colloid in the reference frame. The main results of this paper about the properties of fluctuations and friction in non-equilibrium steady states, as well as the validity and breaking of fluctuation–dissipation relations are presented in Section V. In Section VI we then discuss the implications of these results for future investigations of non-equilibrium systems. We summarize and conclude in Section VII.

## II. Fluctuation–dissipation relations

In this chapter we first review the basic principles of linear response theory to derive the (first) fluctuation–dissipation relation (1FDT). Since this formalism can be found in standard textbooks (see, *e.g.* ref. 13) we keep our discussion to a very minimum, only introducing the fundamental equations that will be important for the results of this work. In the second part we then discuss the generalized Langevin equation and how it can be connected to the second fluctuation–dissipation relation (2FDT), even in non-stationary non-equilibrium situations.

### A. Linear response theory and the 1FDT

The fundamental idea of linear response theory is to determine the time-dependent response function, *χ*(*t*), which defines the response of an observable in the system to an external perturbation, *α*(*t*), of the Hamiltonian, *H* = *H*_0_ − *α*(*t*)*X*. Here, *H*_0_ is the equilibrium Hamiltonian. Under the assumption that *α*(*t*) is a small parameter and the system is in equilibrium for *t* < 0 one can immediately derive the response of an observable *Y*, determined by,1
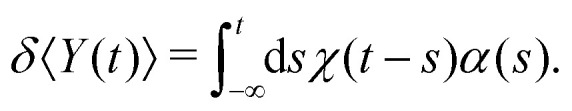
The response function is determined by the 1FDT, which in classical systems can be derived as,2
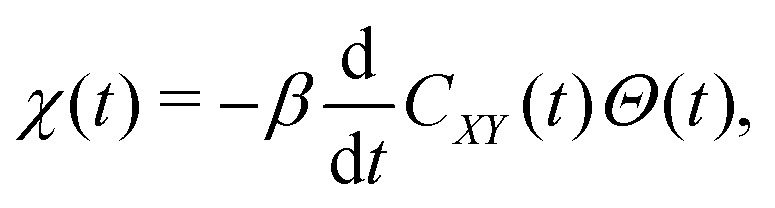
with the Heaviside *Θ* function, where *β* is the inverse temperature *β*^−1^ = *k*_B_*T* and *C*_*XY*_(*t*) is the equilibrium correlation function,

3 *C*_*XY*_(*t*) = 〈*X*(0)* *Y*(*t*)〉_eq_ = 〈*X*(0)|*Y*(*t*)〉,

given by the inner product in the vector space of observables,4
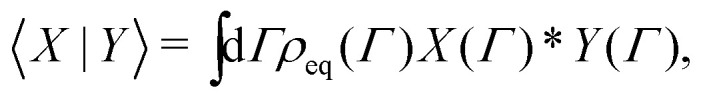
with probability density *ρ*_eq_(*Γ*) defined on the phase space points *Γ* = (*x*,*p*).

In Section VC we apply a perturbation *α*(*t*) = *MV*_0_*δ*(*t*), *i.e.* an instantaneous force, acting on the position of the colloid, *X*, and we investigate the response of the velocity, *Y*(*t*) = *V*(*t*). Here, *M* is the colloid mass and *V*_0_ the instantaneous velocity, *V*_0_ = δ〈*V*(0)〉. The 1FDT can thus be transformed to,5
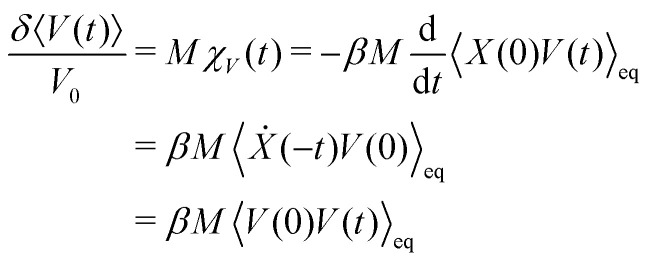
6
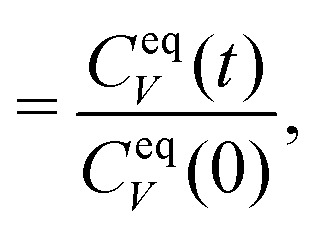
with the velocity auto-correlation function (VACF), *C*^eq^_V_(*t*) = 〈*V*(0)*V*(*t*)〉_eq_. As has been discussed in the literature, under mild assumptions one expects this relationship to hold even in non-equilibrium steady-states if the dynamics are investigated in the colloid frame.^6,17,26^ These assumptions include that the solvent has the same properties as in equilibrium (*i.e.* Boltzmann-distributed velocities according to temperature *T*) which implies that the system is close to equilibrium. In this work, we will apply the perturbation *α*(*t*) in stationary but non-equilibrium systems induced by a permanent external pulling force *F*_ext_ acting on the colloid. The velocity *V*_0_ will be chosen parallel to *F*_ext_. The assumption of being close to equilibrium is thus no longer valid in situations where the external driving on the colloid is strong enough to heat up the surrounding fluid. In this case the equilibrium averages have to be replaced by non-equilibrium averages, *C*^ss^_V_(*t*) = 〈*V*(0)*V*(*t*)〉_ss_, in the stationary state. Throughout this work we will identify the value 
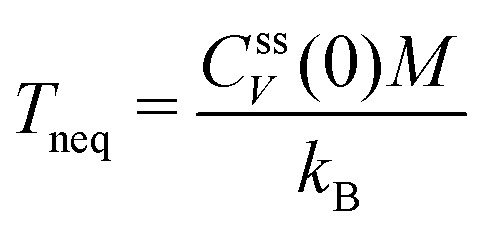
 as the non-equilibrium “temperature” of the fluid. At equilibrium, one clearly has *T*_neq_ = *T* (and thus *β*_neq_ = *β*). In situations close to equilibrium where *β*_neq_ ≈ *β*, eqn (5) can still assumed to be valid.^6^ For larger external driving, eqn (6) is correct for *t* = 0 and it remains to be investigated whether it also holds for larger times *t* > 0.

### B. Generalized Langevin equation and the 2FDT

In active microrheology, one usually solely investigates the motion of the immersed colloid, given by its position and velocities {*X*(*t*),*Y*(*t*)}. The other degrees of freedom in the system, *i.e.* the positions and velocities of the solvent particles are thus not considered, and only affect the motion of the colloid indirectly *via* effective equations of motion. If the microscopic dynamics are Hamiltonian, the Mori–Zwanzig projection operator (MZ) formalism is a powerful tool to derive an exact relation for these effective dynamics.^29,35,36^ The final result is given by the generalized Langevin equation (GLE) for a given set of selected variables {*A*_*i*_},7
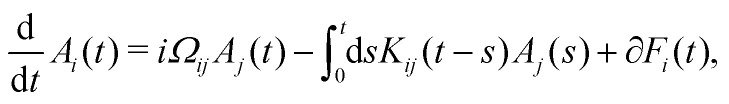
including the frequency matrix *Ω*_*ij*_ that describes direct interactions between the variables *A*_*i*_, the memory kernel *K*_*ij*_(*t*) and the fluctuating force ∂*F*_*i*_(*t*), for which the MZ formalism provides explicit expressions,^29^ but which are very difficult to evaluate analytically in a general framework. From the MZ formalism it is, however, possible to derive a Volterra equation of first kind,8
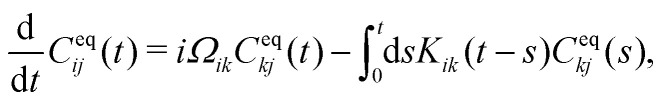
where the correlation function *C*^eq^_*ij*_(*t*) = 〈*A*_*i*_(*t*)*A*_*j*_(0)〉_eq_ is accessible in computer simulations^37^ and experiments.^38^ This Volterra equation thus allows to systematically determine the deterministic parts of the generalized Langevin equation, and it directly follows from the orthogonality condition for the fluctuating force,

9 〈*A*_*i*_(0)∂*F*_*j*_(*t*)〉_eq_ = 0.

The expression for the memory kernel in the MZ formalism can also be transformed into the 2FDT,

10 〈∂*F*_*i*_(0)∂*F*_*j*_(*t*)〉_eq_ = *K*_*ik*_(*t*)〈*A*_*k*_(0)*A*_*j*_(0)〉_eq_,

which, similar to the 1FDT, therefore directly connects the friction interactions in the system with the correlations of fluctuations. We should however note that in general, the MZ formalism does not predict the fluctuating forces to be Gaussian distributed, and indeed, strong deviations from Gaussianity have been observed even in simple equilibrium systems.^37,39^ An important application of the 2FDT is for example the Nyquist relation, which relates the resistance of a resistor to its thermal electric noise.^40,41^ The 2FDT also plays an important role in non-Markovian modeling.^37,38,42^

As has been discussed in the introduction, the validity of this 2FDT in dissipative systems far from equilibrium has been questioned. However, we will now show that it is much more generally valid. For simplicity, we omit direct interactions between selected variables in the following. We consider a set of selected variables *A*_*i*_(*t*) whose dynamical evolution is characterized by a correlation matrix *C*_*ij*_(*t*,*t*_0_) = 〈*A*_*i*_(*t*)*A*_*j*_(*t*_0_)〉_neq_. Here, 〈⋯〉_neq_ denotes the non-equilibrium average over an ensemble of trajectories starting from an initial probability density *ρ*(*Γ*) at an arbitrary “initial” time *T* < *t*,*t*_0_, which can also be chosen *T* → −∞. We do not impose invariance with respect to time translation. However, we assume that the correlation functions can be connected to memory kernels *K*_*ij*_(*t*,*t*_0_) by means of a deterministic Volterra equation11
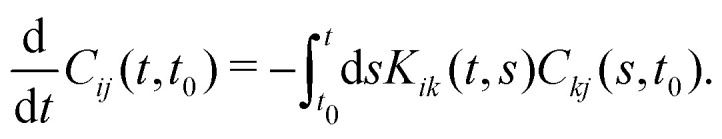
In time-translation invariant systems with *C*_*ij*_(*t*,*t*_0_) = *C*_*ij*_(*t* − *t*_0_), this is certainly true, as one can solve eqn (11) for *K*_*ij*_(*t* − *s*) in a straightforward manner using Fourier methods (with some adjustments in case *Ċ*_*ij*_(0) ≠ 0, see Appendix A). Eqn (11) has been derived for Hamiltonian systems with a time-dependent projection operator formalism by Meyer *et al.*^30^ Here, we take a more general point of view, and see the equation simply as a way to reparametrize the correlation functions *C*_*ij*_(*t*,*t*_0_).

Based on eqn (11), we show in the Appendix A that the correlation structure defined by *C*_*ij*_(*t*,*t*_0_) can be reproduced by a coarse-grained non-stationary GLE model of the form,^30,43^12
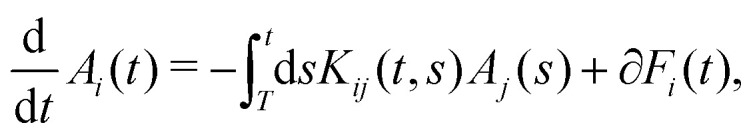
where the Volterra equation automatically implies the 2FDT

13 〈∂*F*_*i*_(*t*)∂*F*_*j*_(*t*_0_)〉_neq_ = *K*_*ik*_(*t*,*t*_0_)*C*_*kj*_(*t*_0_,*t*_0_).

This is a central result of this paper since it states that there is no fundamental violation of the 2FDT in non-equilibrium systems. This statement is not restricted to Hamiltonian systems, and the derivation does not rely on the Mori–Zwanzig formalism. In the following we will therefore refer to it as the second fluctuation–dissipation theorem (2FDT) also in non-equilibrium settings.

It is possible to establish a relation to the Mori–Zwanzig framework by noting (see Appendix A) that eqn (11) is also equivalent to the requirement 〈∂*F*_*i*_(*t*)*A*_*j*_(*T*)〉 = 0 at time *t*_0_ = *T*. Hence the Volterra equation also implies that the fluctuating force is perpendicular to the selected variable *A*_*i*_ at some (arbitrary) reference time *T*. We emphasize that we do not assume the fluctuating force to be Gaussian distributed.

In the following, we will first investigate the implications of this result on the concrete example of active microrheology. We therefore describe the dynamics of the colloid in the colloid frame using the selected variables {*A*_1_ = *X*, *A*_2_ = *V*}, resulting in the GLE,14
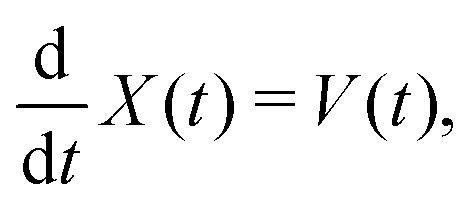
15
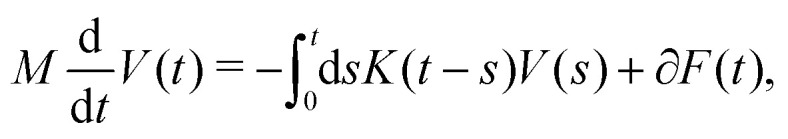
together with the 2FDT,

16 〈∂*F*(0)∂*F*(*t*)〉_ss_ = *k*_B_*T*_neq_*K*(*t*).

The only difference to the equilibrium case is thus the usage of the non-equilibrium temperature *k*_B_*T*_neq_ = *C*^ss^_V_(0)*M*, as defined above.

In recent years several different numerical algorithms have been proposed to calculate the memory kernel from microscopic simulations.^37,39,44,45^ Here, we employ the most straightforward reconstruction technique, directly based on the numerical inversion of the Volterra equation,17
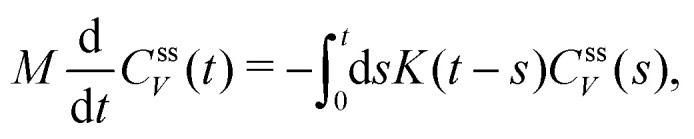
which is the stationary version of eqn (11). Having reconstructed the memory kernel using time correlation functions determined in microscopic trajectories, we can directly use these trajectories to also access the fluctuating force *via* a trivial rewriting of the GLE,18
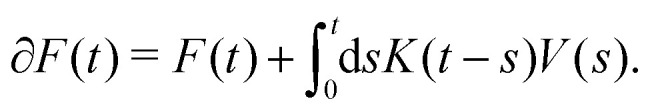
Here, *F*(*t*) is the instantaneous force acting on the colloid, as calculated in the microscopic trajectory. This relations thus allows us to independently and unambiguously verify the validity of the 2FDT. Importantly, it also enables us to access the probability distribution of ∂*F*(*t*).

## III. Computer simulations and modeling

In this work we simulate a colloid immersed in a DPD fluid. In DPD the fluid particles interact *via* dissipative and random pair forces, which are constructed such that the total momentum in the fluid is conserved.^33^ Both forces are connected *via* fluctuation–dissipation relations such that a canonical distribution is reached at equilibrium.^34^ The DPD equations of motion can be written as stochastic differential equations^34^19
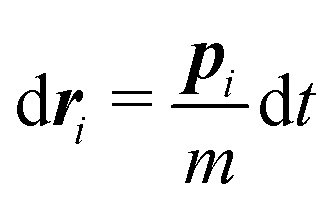
20
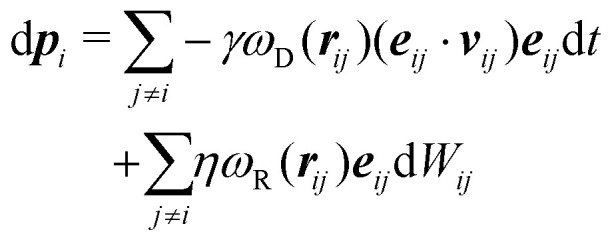
with velocity difference ***v***_*ij*_ = ***v***_*i*_ − ***v***_*j*_, distance ***r***_*ij*_ = ***r***_*i*_ − ***r***_*j*_, connection vector ***e***_*ij*_ = ***r***_*ij*_/|***r***_*ij*_| and the fluctuation–dissipation relations 
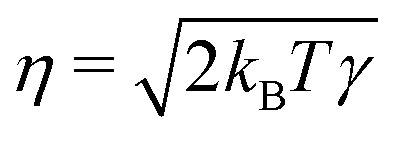
 and *ω*_D_(*r*) = *ω*_R_(*r*)^2^. The random forces are described by independent increments of a Wiener process d*W*_*ij*_d*W*_*i*′*j*′_ = (*δ*_*ii*′_*δ*_*jj*′_ + *δ*_*ij*′_*δ*_*ji*′_)d*t*.^34^ In the present work, we do not include any conservative forces in the DPD equations of motion. Since DPD is purely based on pairwise interactions, it can be regarded as a Galilean invariant thermostat. Marsh *et al.*^46^ showed that DPD indeed reproduces the hydrodynamic equations (Navier–Stokes equation) and calculated theoretical values for transport coefficients.

Here, we use the weight function 
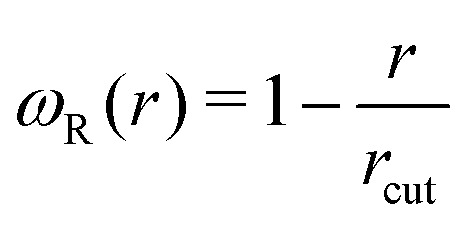
. The simulation units are given by *k*_B_*T* = *ε* (unit of energy), *r*_cut_ = *σ* (unit of length length) and 
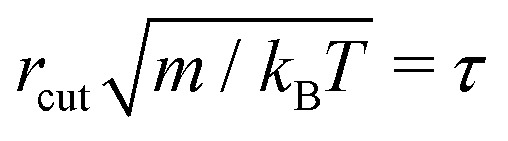
 (unit of time). We choose the DPD parameters, *γ* = 5*ετσ*^−2^, density *ρ* = 3*σ*^−3^ and the time step Δ*t* = 0.005*τ*. The shear viscosity is *η* = 1.28*ετσ*^−3^ ^47^ and the solvent diffusion coefficient can be approximated to *D*_s_ = 0.75*σ*^2^*τ*^−1^.^46^

The colloid is modelled as a raspberry-like object, consisting of 80 independent particles placed on a spherical shell with radius *R* = 3*σ*. The total mass of the colloid is *M* = 80*m*. The colloid is a rigid body, *i.e.*, the relative distances of all particles forming the colloid are fixed. These particles interact with the fluid particles *via* a purely repulsive interaction, *i.e.*, a truncated LJ potential with cutoff 
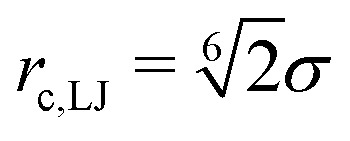
. We use a cuboid simulation box with periodic boundary conditions in all three dimensions and edge lengths *L*_*x*_ = 55.4689*σ*, *L*_*y*_ = *L*_*z*_ = 27.7345*σ*. To create the non-equilibrium steady-state we pull on the colloid with a constant and permanent force *F*^ext^ in positive *x*-direction, and apply a very small negative bulk force to the fluid such that the total momentum in the system is conserved. All simulations are performed using the simulation package Lammps.^48,49^

To determine the response δ〈*V*(*t*)〉 and test the 1FDT in computer simulations, we apply the perturbation *α*(*t*) = *MV*_0_*δ*(*t*) to a steady-state system. We then perform two simulations in parallel; one with the perturbation (pert) and one without (unpert). The response can then be calculated as δ*V*(*t*) = *V*_pert_(*t*) − *V*_unpert_(*t*). This quantity is then averaged over many different systems, δ〈*V*(*t*)〉, with initial perturbations at *t* = 0. With this method, some statistical noise in the calculation of the response function can be eliminated.

## IV. Linear and non-linear response in active microrheology

In this section we analyze the response of the colloid to the permanent external force, *F*^ext^. After applying the force we simulate sufficiently long that the system reaches a steady state. All quantities that will be reported in the following are averages in these non-equilibrium steady states.

### A. Linear response

**Fig. 1:**
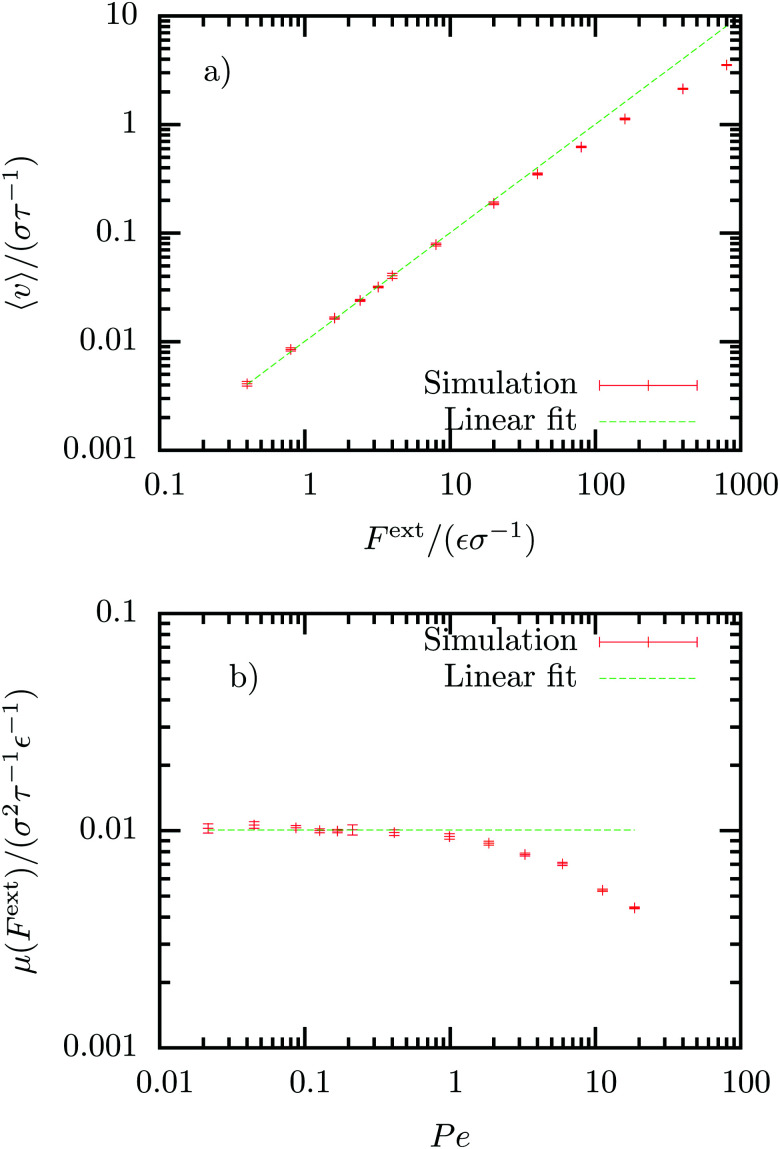
Response of colloid to an external force *F*^ext^. in non-equilibrium steady-state. (a) Average velocity 〈*v*〉. (b) Force-dependent mobility 
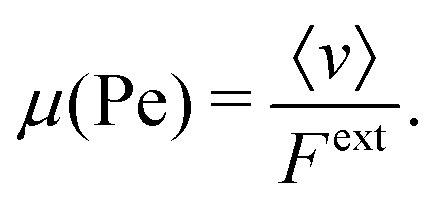
, plotted against Peclet number. The data was fitted in the linear regime, *F*^ext^ = [0,10], revealing an equilibrium mobility *μ* = (0.0101 ± 0.0001)*σ*^2^*ε*^−1^*τ*^−1^.

For small external forces we observe an extended linear response regime, in which the average steady-state velocity is given by, 〈*v*〉 = *μF*^ext^, with constant mobility *μ* (see Fig. 1a). Using the linear response regime, we can extract the mobility *μ* = (0.0101 ± 0.0001)*σ*^2^*ε*^−1^*τ*^−1^. An estimate of the mobility can also be determined using linear response theory, by integration of the VACF21
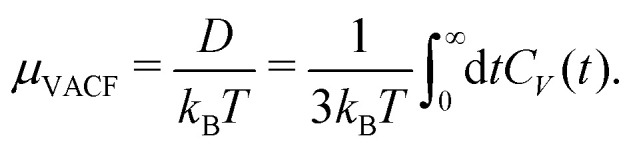
and by integration over the memory kernel which appears in the generalized Langevin equation,22
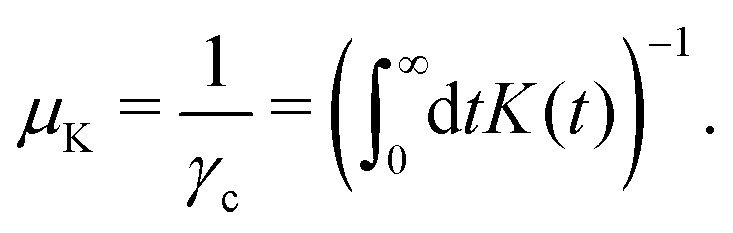
The results for these dynamic correlation functions will be discussed later (see Section VB and Fig. 4). Extracting the mobility from these quantities results in *μ*_VACF_ = (0.0103 ± 0.0001)*σ*^2^*ε*^−1^*τ*^−1^ and *μ*_K_ = (0.0113 ± 0.0004)*σ*^2^*ε*^−1^*τ*^−1^, in good agreement with the mobility determined above.^50^ The discrepancy in the value *μ*_K_ most probably arises from the memory reconstruction which becomes less accurate for longer times. Using Fourier transform techniques in the long-time regime as described in ref. 51 and 52 might improve these values.

Using the solvent diffusion coefficient, *D*_s_, we can also define the Peclet number, Pe = 〈*v*〉/*v*_diff_, with 
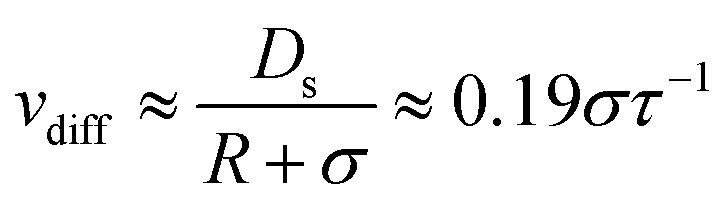
. This dimensionless quantity thus quantifies the ratio of the advective transport due to the external force to the diffusive transport. The linear response regime extends to Peclet numbers of roughly Pe < 1, as can be observed in Fig. 1b. For larger driving forces, the mobility clearly depends on the strength of the external force.

### B. Beyond linear response: thickening

Beyond the linear response regime, different non-linear behaviours have been observed, including thinning in Brownian suspensions^32,53^ and glass-forming Yukawa fluids,^54,55^ as well as thickening in granular systems.^32,56,57^ Both thinning and thickening behaviour has been observed in a model colloidal system with solvent particles described by a Langevin equation.^58^ The authors explain this with the transition from a diffusive to a damping regime at low Peclet numbers and from a damping to a collision regime at high Peclet numbers. In our simulations using a dense DPD fluid, we do not observe a thinning regime, but directly thickening at Pe > 1, (see Fig. 1b).

## V. Structure, fluctuations and dissipation in the colloid frame

Having analysed the velocity which the colloid attains in the non-equilibrium steady state we will now study its properties in the colloid frame. This includes density and velocity profiles of the surrounding fluid, as well as validations of the two fluctuation–dissipation relations as introduced in Section II.

### A. Radial distribution function and velocity profiles

**Fig. 2:**
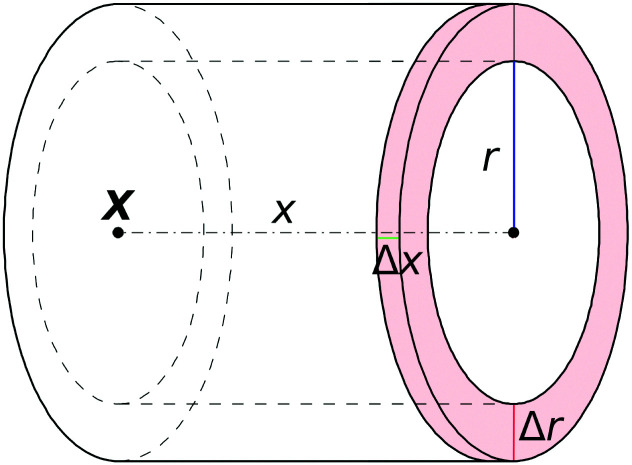
Cylindrical geometry in which the profiles in Fig. 3 are evaluated. The colloid center is ***X***. In this work we have used Δ*r* = 0.2*σ* and Δ*x* = 0.4*σ*.

**Fig. 3:**
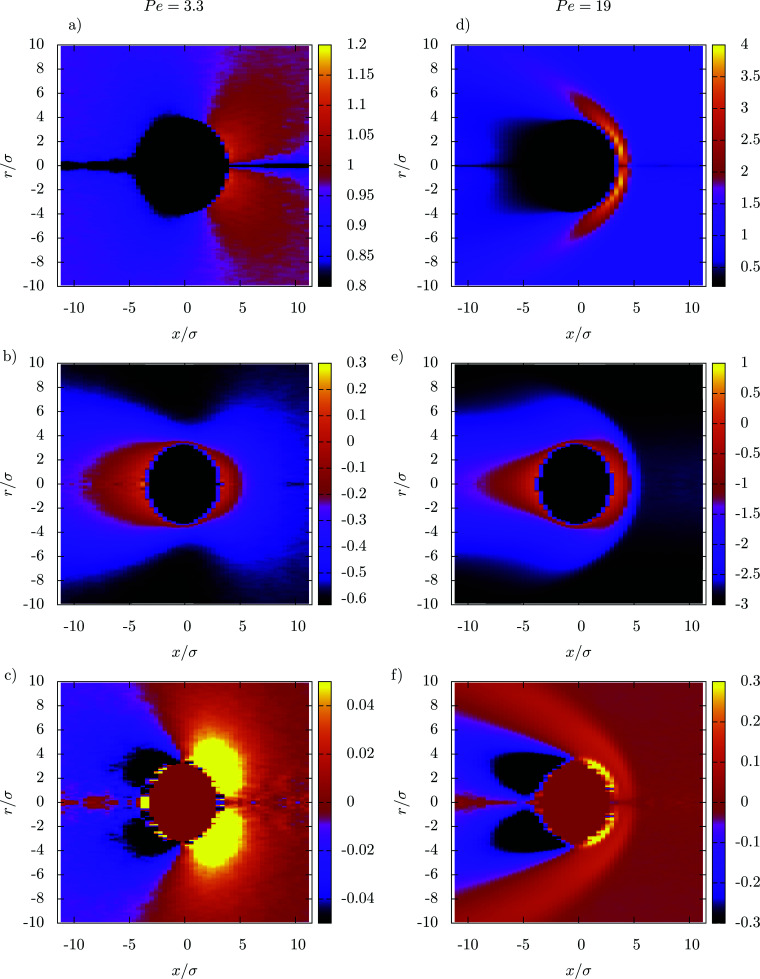
Radial distribution function and velocity profiles of DPD particles around the colloid in non-equilibrium steady-states with different Peclet numbers Pe. The quantities are calculated in a cylindrical geometry where the axis is set by the direction of the external force (pulling in positive *x*-direction). Averages are taken in wheel-shaped slices with radius *r* and a mid-point distance *x* from the colloid center (see Fig. 2). For the sake of visualization, the profiles are mirrored at *r* = 0, *i.e. f*(−*r*) = *f*(*r*). Shown are radial distribution function *g*(*x*,*r*) (a and d), average velocity parallel (b and e) and perpendicular (c and f) to the direction of the external field. The stripe at |*r*| < 0.2*σ* results from insufficient sampling in a very small volume. The colour code shows the magnitude of the scalar fields (different scales for each plot).

To quantify the density profile around the colloid we calculate the radial distribution function,23
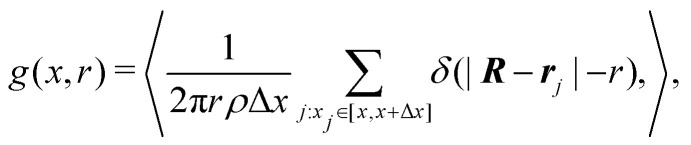
using the cylindrical geometry sketched in Fig. 2. Depending on the Peclet number, the radial distribution functions behave qualitatively different (see Fig. 3a and d). While for smaller Peclet number the structural deformation still reminds of a diffusive dipole^59^ (albeit Pe is already relatively large in Fig. 3a) the structure is completely different for large Pe in which a significant bow wave emerges and a wake with much fewer particles trails the colloid. This quantitative difference between Pe ≲ 3.3 and Pe ≳ 18.6 is also perfectly visible in the velocity profiles. While for small Pe the profiles still display a certain symmetry between the front and the back of the colloid (see Fig. 3b and c), for higher Pe this symmetry is broken. In particular, the bow wave in front of the colloid is nicely visible in Fig. 3e and the wake behind the colloid in Fig. 3f.

From this analysis we can thus conclude that although the linear response regime only extends up to Pe < 1, the properties of the surrounding fluid significantly changes only for larger Pe > 3.3. We therefore expect that this similarly holds for the dynamic properties of the colloid in the colloid frame.

### B. Dynamic correlations and memory kernels

**Fig. 4:**
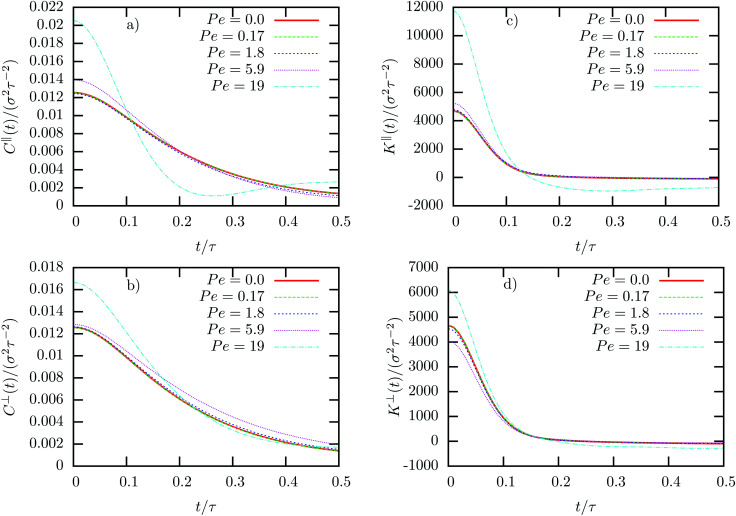
Velocity auto-correlation function *C*(*t*) and memory kernel *K*(*t*) of colloids in non-equilibrium steady-states with different Peclet numbers Pe. Velocity fluctuations and memory kernel are calculated in the colloid frame parallel (a and c) and perpendicular (b and d) to the direction of the external force.

The velocity auto-correlation function (VACF) of the colloid in the colloid frame is shown in Fig. 4. Without external driving, the VACF is governed by an initial exponential decay, followed by the usual hydrodynamic ling-time tail, which can be described by the power law *C*_V_(*t*) ∼ *t*^−3/2^ ^60,61^ (not shown here due to large statistical fluctuations). As expected from linear response theory, the correlation functions for Pe < 1 are independent of the external force and isotropic. When increasing the driving above Pe > 1 the first small deviations are observable for larger times, which are, however, barely visible, in agreement with our previous observations. For very large Peclet number the VACF then qualitatively changes. First, we observe an increase in “local temperature” as defined kinetically *via T*_neq_ ∼ *C*_V_(*t* = 0). Second, the changes of the local fluid structure induce an oscillatory behavior in the VACF parallel to the external driving, *C*^‖^(*t*), as can be seen in Fig. 4a. If the colloid moves in the negative *x*-direction, it leaves the bow wave which counteracts the external driving, which automatically means that the external force will push the colloid back into position. If the colloid moves in the positive *x*-direction, the density of fluid particle significantly increases which similarly leads to a restoring force. Both cases thus effectively induce a “trapped” motion, which explains the oscillations in the VACF. In perpendicular direction, this effect is much smaller, most importantly, the local temperature does not increase as much as in parallel direction, *i.e. T*^⊥^_neq_ < *T*^‖^_neq_ (see Fig. 4b). Moreover, in the intermediate driving regime at Pe = 5.9 one in fact observes a slower decay of the VACF, which is most probably due to the small decrease in density in the direction perpendicular to the colloid. Only when increasing the driving even further, one observes a similar behavior as discussed in parallel direction, consistent with the change in structure shown in Fig. 3a and d.

The same observations hold for the memory kernel *K*(*t*). In equilibrium, the memory kernel also has an initial exponential decay, however for larger times it becomes negative and approaches zero with the same power law as the VACF but different sign, 
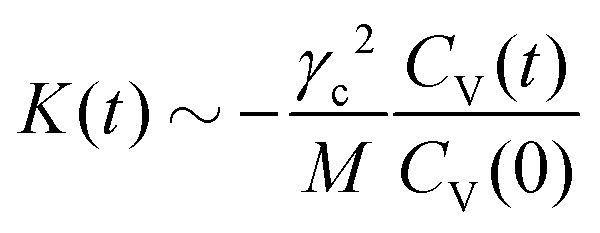
.^52,62^ The oscillatory dependence on *t* discussed in the VACF for large Pe is reflected in the memory kernel by a strong initial damping, followed by a very pronounced minimum with negative friction (see Fig. 4c and d).

### C. Violation of 1FDT for strong external driving

**Fig. 5:**
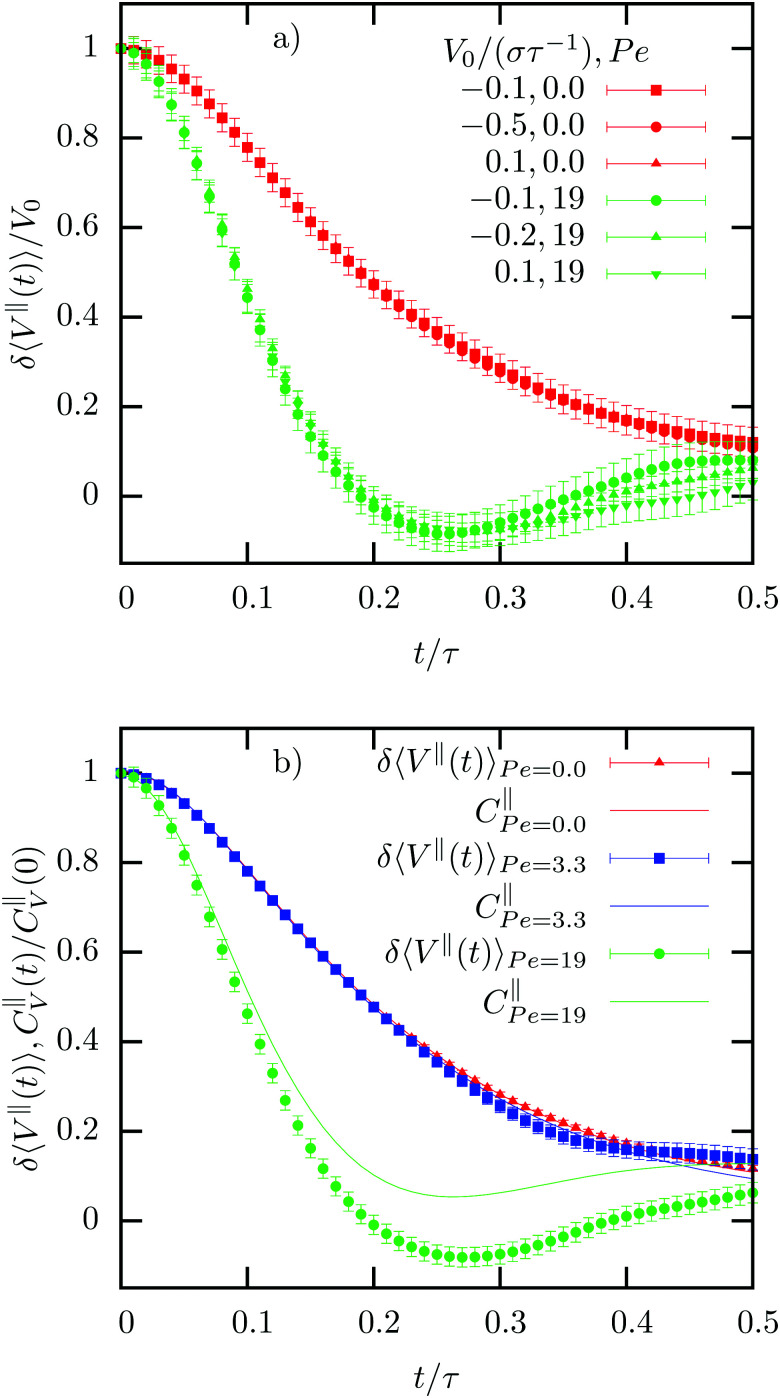
Non-equilibrium response to a force impulse at time *t* = 0 in non-equilibrium steady-states with different Peclet numbers Pe. The perturbation *α*(*t*) = *MV*_0_*δ*(*t*) is applied parallel to the permanent external force. (a) Response δ〈*V*^‖^(*t*)〉, for different values of the initial velocity difference *V*_0_. (b) Response compared to the normalized velocity auto-correlation function *C*^‖^_V_(*t*)/*C*^‖^_V_(0), evaluated in steady-state.

In the colloid frame, we can also calculate the non-equilibrium response δ〈*V*^‖^(*t*)〉 of the colloid to a perturbation induced by a force impulse at time *t* = 0 in the positive *x*-direction, as defined in Section IIA. We emphasize that this force impulse is applied in addition to the permanent external force *F*_ext_. Hence we consider a time-dependent perturbation in a stationary non-equilibrium state in which the colloid moves with a constant velocity, driven by the permanent external force. In equilibrium systems, according to the linear response theory, this response depends linearly on the amplitude of the perturbation. As can be seen in Fig. 5a, the normalized response is independent of the strength and direction of the force impulse (*i.e.* parallel or anti-parallel to the external driving of the colloid), which shows that this expectation is still fulfilled at nonequilibrium.

Comparing this normalized response to the VACF, we can immediately investigate the validity of the 1FDT. While in equilibrium the 1FDT is indeed fulfilled, for strong external forces we can clearly observe a strong violation of the first fluctuation–dissipation relation. To rationalize this important observation, we recall the results of the previous section. There, we have discussed that the instantaneous fluctuations of the velocity, *C*_V_(0), in the directions parallel and perpendicular to the external force are significantly different. A closer look at the bow wave in Fig. 3d–f also shows that the structure in the surrounding fluid can induce a coupling between these two different directions. This means that the effective restoring forces, *F*^‖^(*x*,*r*), which induce the oscillatory behavior of *C*^‖^_V_(*t*) at strong driving, do not only depend on *x*, but also on *r* (and similarly in perpendicular direction). We therefore have precisely the situation described in the introduction, with two coupled degrees of freedom which have different temperature.^27,28^ Here, the situation is clearly more complicated than in this toy model and it is not possible to disentangle the different contributions to the response function, but the model gives a reasonable explanation for the (apparent) violation of the 1FDT in active microrheology.

### D. Thermal fluctuations and 2FDT

Having investigated the first fluctuation–dissipation relation, we now use the methodology described above (see Section IIB) to determine the thermal fluctuations in active microrheology and thus the second fluctuation–dissipation relation.

**Fig. 6:**
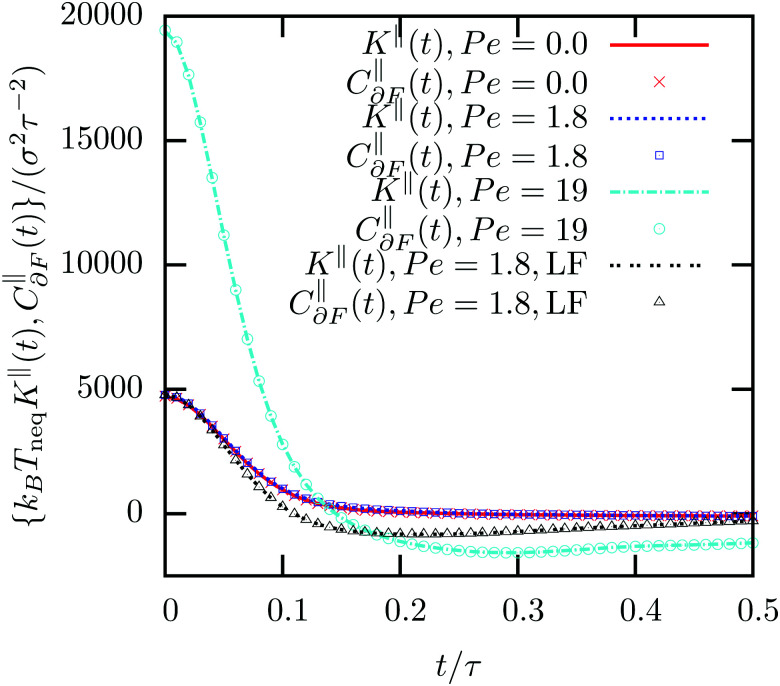
Memory kernels, *k*_B_G*T*_neq_*K*^‖^(*t*), and auto-correlation function of the stochastic force, *C*^‖^_∂*F*_(*t*), in non-equilibrium steady-states with different Peclet numbers Pe. The last two curves (black) correspond to the application of *t*he memory kernel formalism in the laboratory frame (LF).

Comparing the time-correlation function of the thermal forces, *C*_∂*F*_(*t*) = 〈∂*F*(0)∂*F*(*t*)〉_ss_ with the memory kernel *K*(*t*) discussed at the beginning of this section, we clearly see that the 2FDT is fulfilled for all different driving forces (see Fig. 6). Different from the 1FDT, which only holds strictly in equilibrium conditions (at least in its naive version, as discussed in ref. 27), the 2FDT indeed remains valid in a non-equilibrium steady state. This numerically confirms the theoretical calculations presented in Section II and derived in Appendix A. Interestingly, one can also calculate the correlation functions in the laboratory frame and extract the memory kernel and the thermal fluctuations in the same way as described before. The resulting memory kernel will clearly be different, but the validity of the 2FDT is not affected (see Fig. 6, black curve). This result also highlights the importance of describing the system in the colloid frame. While both descriptions are mathematically sound, the description in the colloid frame highlights the universality of the memory kernel inside the linear response regime (and even beyond).

**Fig. 7:**
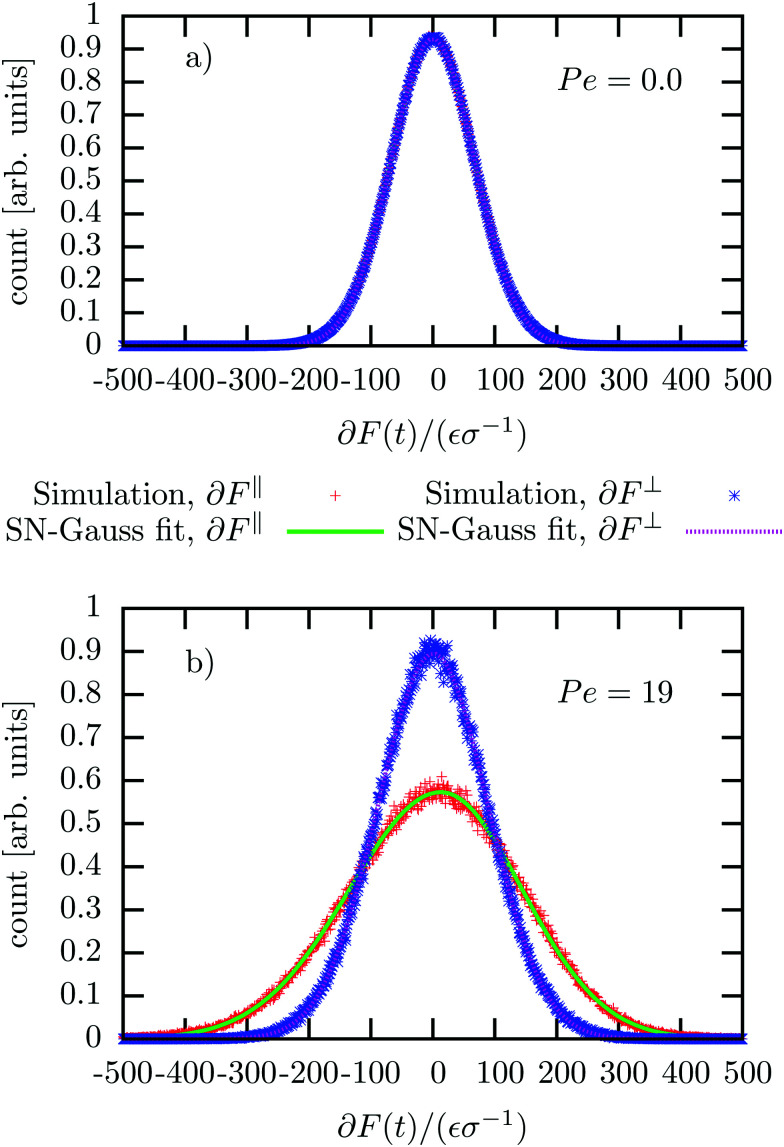
Distribution of the stochastic force, ∂*F*(*t*) in non-equilibrium steady-states with different Peclet numbers, Pe = 0.0 (a) and Pe = 19 (b). The data is fitted with a split normal distribution (see eqn (24)) (SN-Gauss). In the top panel, all curves perfectly overlap.

We also investigate the distribution of the thermal fluctuations. In equilibrium, the distribution is an almost perfect Gaussian function, as one might expect from central limit theorem (see Fig. 7a), since the total force consists of hundreds of collisions which are basically independent (apart from hydrodynamic interactions). Interestingly this no longer holds outside the linear response regime. In Fig. 7b one can clearly observe a slight asymmetry in the distribution of forces parallel to the external driving, which occurs due to a long tail of “negative forces” (*i.e.* anti-parallel to the external force). This tail emerges since the colloid is constantly pulled through an otherwise stationary fluid. Inside the linear response regime, the diffusive dipole as discussed in ref. 59 ensures that the particles close to the colloid indeed have the same relative velocity (and thus the same statistics of collisions). This is no longer the case for Pe > 1, hence some fluid particles, illustratively, crash into the colloid, and thus induce large negative forces. Since the total average force is zero, these strong negative forces have to be compensated by a slightly enhanced probability of observing a positive thermal force. The distribution outside the linear response regime can, in fact, be described by a split normal distribution (SN-Gauss),24
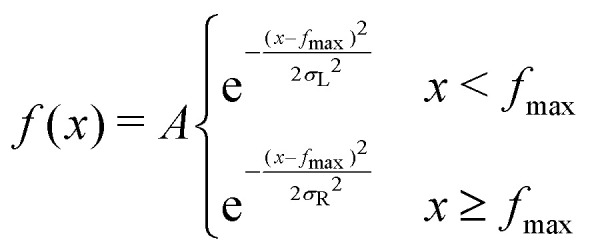
with mean *f̄* = 0 and maximum 
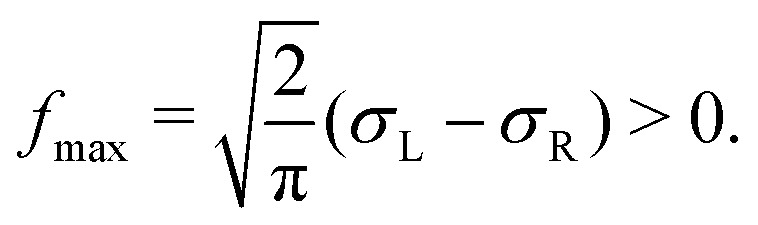


**Fig. 8:**
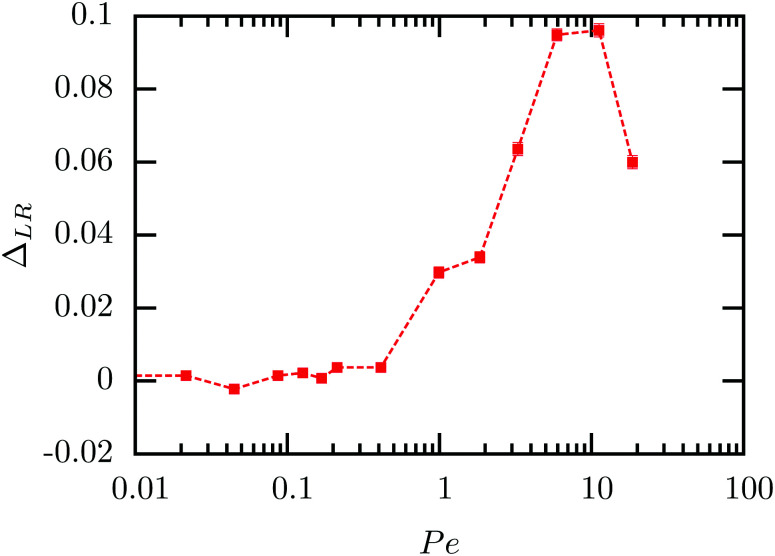
Asymmetry factor *Δ*_LR_ of the split normal distribution as fitted in Fig. 7. The error bars are smaller than or basically have the same size as the points.

Using the split normal distribution, we can define an asymmetry factor 
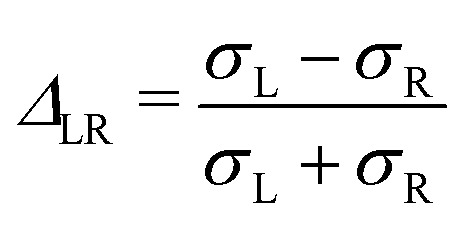
. It shows an unexpected non-monotonic dependence on the Pe number (see Fig. 8). For Pe < 1 it is clearly zero, consistent with the above discussion of the linear response regime. Intriguingly, we can observe a very sharp transition away from *Δ*_LR_ = 0, allowing us to determine the end of the linear response regime with much more precision than possible from a simple inspection of the average steady-state velocity. Furthermore, the asymmetry reaches a maximum at around Pe = 5 and then decays rapidly again. We explain this behavior with the formation of a thick and dense particle “shield” as illustrated in Fig. 3. This bow wave is dense enough to efficiently accelerate the particles in front of the colloid, shielding it from stronger impacts, as described above.

## VI. Discussion

In this paper we have investigated the validity and potential violations of fluctuation–dissipation relations in a driven system far from equilibrium. We found that the 1FDT is only valid under very restrictive conditions. On the other hand, we provided a mathematical argument and numerical evidence that the 2FDT is exactly fulfilled for all values of driving forces, even far beyond the non-linear response regime.

As mentioned in the introduction, violations of the 2FDT have repeatedly been reported in the literature.^19–22,31^ The reason is that, when investigating the forces on a selected probe particle due to an orthogonal bath, it is not *a priori* clear how to distribute them between the memory and the noise term in the GLE, without additional requirements. Mitterwallner *et al.*^23^ have recently pointed out that an infinite number of GLEs are compatible with a given VACF *C*^ss^_V_(*t*). However, if one imposes an orthogonality condition on the noise or, equivalently, the validity of the Volterra equation, this singles out one GLE, in which the 2FDT is fulfilled.

This remains correct in non-stationary situations, as discussed in Section IIB and should also be valid in the presence of (external) drift terms, *i.e.*, for GLEs of the form25
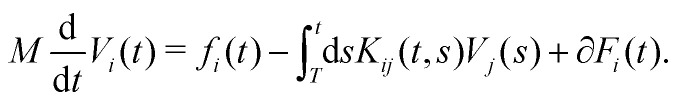
For a given ensemble of trajectories, it can be constructed *via* the following steps:

(1) Determine *V*^0^_*i*_(*t*) = 〈*V*〉^neq^_*i*_(*t*).

(2) Rewrite *V*_*i*_(*t*) = *V*^0^_*i*_(*t*) + *u*_*i*_(*t*), determine *C*^*u*^_*ij*_(*t*,*t*_0_) = 〈*u*_*i*_(*t*)*u*_*j*_(*t*_0_)〉_neq_ and then eqn (25) can be separated as follows26
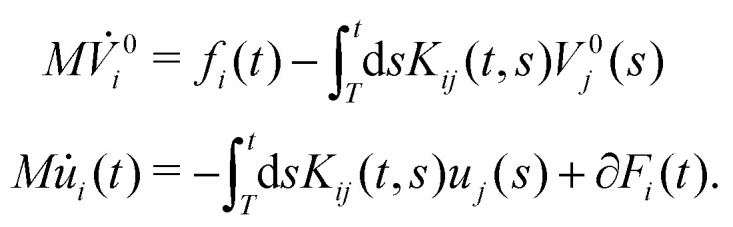


(3) Determine *K*_*ij*_(*t*,*t*_0_) by Volterra inversion of *C*^*u*^_*ij*_(*t*,*t*_0_). One obtains a GLE for *u*_*i*_(*t*) that satisfies the 2FDT.

(4) Determine the effective drift force *via*
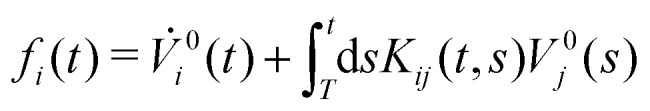
.

The resulting GLE would satisfy the 2FDT.

Cui *et al.* have discussed a particularly intriguing case of a particle coupled to a bath of charged oscillators and subject to an oscillatory electric field.^22^ They derived a GLE by integrating out the bath particles following a procedure outlined by Zwanzig.^29^ The resulting GLE does not satisfy the 2FDT, moreover, the noise has a deterministic component that reflects the oscillatory motion of the charged bath particles. Based on our construction above we argue that also in this system, an equivalent GLE can be constructed that does satisfy the 2FDT.

To summarize, it is possible to formulate GLEs that do not satisfy the 2FDT. It some cases, working with them may be more convenient – they may have a simpler structure, the simulation may be easier, or they are the direct result of a theoretical calculation.^22,66^ However, different from the 1FDT one cannot use such equations to postulate a fundamental violation of the 2FDT, as it is always possible to construct equivalent GLEs that do satisfy the 2FDT. If the GLE is constructed based on the principle that the noise should be perpendicular to the selected variable at some time *t* = *T*, then this automatically results in the 2FDT relation. From a modelling perspective this latter choice strikes us as desirable since it enables a systematic and unique separation into deterministic drift, deterministic memory and friction forces as well as stochastic noise, as illustrated above.

## VII. Conclusion

In this work we have investigated the dynamical properties of colloids in a system far from equilibrium, in which a colloid is pulled with a constant force through a fluid. First, we have identified the linear response regime and characterized the shear thickening behaviour of the suspension when driving the colloid beyond linear response. Second, we have investigated dynamic properties in the Galilean reference frame which moves with the average velocity of the colloid. We were thus able to characterize in detail the impact of the non-equilibrium conditions on the dynamic correlation functions, the memory kernels and the FDTs.

With our analysis, we have observed a violation of the 1FDT, *i.e.* the relationship between non-equilibrium response and the stationary correlation function. The violation can be explained by the emergence of two different “temperatures” in the direction parallel and perpendicular to the external driving. Furthermore, we have validated the 2FDT, *i.e.* the connection between the friction and stochastic interaction in the system, even in conditions far away from equilibrium. We have further studied the properties of the stochastic forces and found an emerging asymmetry in its distribution function, which can be described by a screw normal distribution. This asymmetry appears to be a strong indicator to determine the linear response regime, since it depends very sensitively on perturbations of the usual diffusive dipole.^59^

The purpose of this work is to engage a discussion on fluctuation–dissipation relations, particular the 2FDT, in out-of-equilibrium conditions. As we have argued in the previous section and as has been argued by Mitterwallner *et al.*,^23^ the distinction between systematic and stochastic interactions with bath particles is *a priori* somewhat arbitrary.

We therefore suggest to impose, as additional fundamental criterion, an orthogonality condition as it directly follows from the Mori–Zwanzig formalism.^29,30^ This uniquely defines the relationship between the dissipative and the random forces in the system, which is then given by the 2FDT, and it is applicable to systems far away from equilibrium and also non-stationary dynamics. Such a separation is crucial for consistent modeling and should enable to use dynamic coarse-graining techniques developed in recent years^42,63,64^ for non-equilibrium systems. From a practical point of view, it could sometimes be convenient to use equivalent versions of the GLE that violate the 2FDT. However, in our opinion, this should then be seen as a mathematical trick rather than a fundamental property of the underlying dynamical system.

## Conflicts of interest

There are no conflicts to declare.

## Appendices

### Appendix A Volterra equation, orthogonality, and 2FDT in generalized Langevin equations

We consider a general non-stationary GLE of the form

A1

where the memory kernel *K*_*ik*_(*t*,*s*) is not required to be invariant with respect to time translation. Likewise, the correlation function *C*_*ij*_(*t*,*t*_0_) = 〈*A*_*i*_(*t*)*A*_*j*_(*t*_0_)〉_neq_ is not assumed to necessarily depend on (*t* − *t*_0_) only. The time *T* is some arbitrary “initial” time, *T* < *t*, which can also be chosen *T* → −∞.

In the following, we will discuss the connection between the three following relations:

(I) The deterministic Volterra equation

A2

(II) An expression for the correlation between the random force and velocity

A3

for all time pairs (*t*,*t*_0_)

(III) The second fluctuation–dissipation relation

A4 〈∂*F*_*i*_(*t*)∂*F*_*j*_(*t*_0_)〉_neq_ = *MK*_*ik*_(*t*,*t*_0_)*C*_*kj*_(*t*_0_,*t*_0_)

The relation (I) and (III) have been shown to hold for non-stationary Hamiltonian systems using the Mori–Zwanzig formalism.^30,65^

We can easily see that (I) and (II) are equivalent. We simply multiply eqn (A1) with *A*_*j*_(*t*_0_) and take the ensemble average. We will now show that (I) implies (III). To this end, we first express ∂*F*_*j*_(*t*_0_) using eqn (A1) multiply with ∂*F*_*i*_(*t*), take the ensemble average and thus write the force-force correlation as

A5

For the first term on the right hand side we find

A6

which is obtained by taking the derivative of (II) with respect to *t*_0_. The second term can be rewritten as

A7

Here, we have used (II) in the first step, the symmetry property *C*_*ij*_(*s*′,*s*) = *C*_*ji*_(*s*,*s*′) in the second step and (I) in the last step. Combining eqn (A5)–(A7), we finally obtain the fluctuation–dissipation relation (III). Hence the second fluctuation–dissipation relation is a necessary consequence of the deterministic Volterra equation. We emphasize that this is a general relation, which does not rely on the Mori–Zwanzig formalism.

Based on these general results, we can now specifically discuss stationary GLEs with *K*_*ij*_(*t*,*s*) = *K*_*ij*_(*t* − *s*) and their stationary solutions with *C*_*ij*_(*t*,*t*_0_) = *C*_*ij*_(*t* − *t*_0_). We consider the two most popular such GLEs, the Mori–Zwanzig GLE with *T* = 0, and the “stationary GLE” with *T* → −∞.^37^ In these cases, the conditions (I–III) read

A8

(II) (Mori–Zwanzig GLE): 〈*A*_*i*_(0)∂*F*_*j*_(*t*)〉_ss_ = 0.

(II′) (Stationary GLE):^37^

A9

(III) 〈∂*F*_*i*_(0)∂*F*_*j*_(*t*)〉_0_ = *K*_*ik*_(*t*)*C*_*kj*_(0)

We close with a note on the invertibility of eqn (A8). Provided *C*_*ij*_(*t*) has a locally integrable second derivative, eqn (A8) can be inverted in a straightforward manner by Fourier methods, yielding a unique memory kernel *K*_*ij*_(*t*). Whereas this integrability condition is usually met at *t* > 0, it can broken at *t* = 0 if *Ċ*_*ii*_(*t*) ≠ 0 at *t* → 0^+^ (since *Ċ*_*ii*_(*t*) = −*Ċ*_*ii*_(−*t*)). However, such cases can be handled as well. The memory kernel then acquires a δ-shaped instantaneous friction contribution.
